# Effects of Melatonin Administration to Pregnant Ewes under Heat-Stress Conditions, in Redox Status and Reproductive Outcome

**DOI:** 10.3390/antiox9030266

**Published:** 2020-03-23

**Authors:** Efterpi Bouroutzika, Dimitrios Kouretas, Serafeim Papadopoulos, Aristidis S. Veskoukis, Ekaterini Theodosiadou, Sotiria Makri, Charilaos Paliouras, Marios-Lazaros Michailidis, Mariangela Caroprese, Irene Valasi

**Affiliations:** 1 Veterinary Faculty, University of Thessaly, 43100 Karditsa, Greece; bouroutz@uth.gr (E.B.); etheodosiadou@uth.gr (E.T.); chpaliou@uth.gr (C.P.); mamichailidis@uth.gr (M.-L.M.); 2 Department of Biochemistry and Biotechnology, University of Thessaly, Viopolis, Mezourlo, 41500 Larissa, Greece; dkouret@uth.gr (D.K.); veskoukis@uth.gr (A.S.V.); sotirina_m@hotmail.com (S.M.); 3 Department of Ichthyology and Aquatic Environment, University of Thessaly, Fytokou str., 38446 Volos, Greece; serpapad@uth.gr; 4 Department of Sciences of Agriculture, Food and Environment (SAFE), University of Foggia, Via Napoli 25, 71122 Foggia, Italy; mariangela.caroprese@unifg.it

**Keywords:** ewes, heat stress, melatonin, reproductive performance, redox status, milk yield

## Abstract

Heat stress is a known promoter of reactive oxygen species generation, which may compromise pregnancy and foetal development. Melatonin is a pleiotropic molecule that regulates various processes including pregnancy. Thus, it could be used to ameliorate the redox status of pregnant heat-stressed ewes and the outcome of their pregnancy. Sixty-eight ewes participated in the study, which were allocated into two equal groups, i.e., Melatonin (M) and Control (C) group. All ewes were exposed to heat stress from D0 to D120. In both groups, after oestrus synchronization of ewes, rams were introduced to them for mating (D16). In M group, starting with sponges’ insertion (D0), melatonin implants were administered four-fold every 40 days. Pregnancy diagnosis was performed by means of ultrasonography. Daily evaluation of temperature humidity index (THI), rectal temperature, and breathing rate were performed throughout the study. Blood samples were collected repeatedly from D0 until weaning for assaying redox biomarkers. Milk yield was measured thrice during puerperium. The results showed that melatonin administration throughout pregnancy improved the redox status of heat-stressed ewes and increased the mean number and bodyweight of lambs born per ewe, as well as the milk production. Therefore, melatonin may be used as antioxidant regimen in heat-stressed ewes for improving their reproductive traits.

## 1. Introduction

During pregnancy, in sheep, both the pregnant ewes and the foetuses are exposed to oxidative stress caused by the increased amount of reactive oxygen species (ROS) [1,2]. ROS play a crucial role during pregnancy, as they are associated with embryo development, implantation, and defensive functions, as well as with steroidogenesis, pregnancy maintenance, and normal lambing [3]. Various factors, among them heat stress, may lead to increased ROS production [4,5], which may cause the abortion or development of abnormalities in the foetus [6]. Moreover, heat stress may also adversely affect milk quantity and quality [3].

Heat stress is defined as an environment that acts to driving body temperature above a set-point temperature. High ambient temperature, with high direct and indirect solar radiation, wind velocity and relative humidity cause the effective temperature of the environment to often exceed the thermoneutral zone of animals (5–25 °C; [7]), leading to heat stress [8,9]. Heat stress is a significant limiting factor for dairy production in hot climates [10]. Heat-stressed animals show reduced feed and water intake, which may in turn alter their endocrinological profile, thereby increasing the energy requirements for maintenance leading to negative impact in the production and reproductive performance of livestock [11,12]. With regard to reproduction, heat stress can have an impact in ROS generation and consequently in pregnancy progress and foetal development. In female mice, heat stress has resulted in increased ROS activity in the oviducts and the embryos and in reduced glutathione content in the embryos [13,14].

Melatonin is an indolamine with pleiotropic bioactivities [15]. It is a broad-spectrum antioxidant, free radical scavenger and an anti-inflammatory molecule [16,17,18,19] that regulates a number of physiological processes in sheep. With respect to reproduction, melatonin is involved in follicular development, luteal function, and early embryonic development [20,21]. In in vitro conditions, it has also been found to support embryonic development [22]. In a preliminary study, we found that melatonin administration in heat-stressed ewes during the peri-conception period led to increased total antioxidant capacity and improved fertility rate (unpublished results), which points out a potential result of the antioxidant effect of melatonin at follicular level and/or early embryonic development.

Currently, the administration of melatonin to ewes is an established method for accelerating the onset of the breeding period [23]. However, the role of exogenous melatonin in redox regulation and pregnancy outcome in ewes has not been studied so far. The general objective of this study was to evaluate the effects of melatonin administration to pregnant heat-stressed ewes, with respect to the redox status of the animals and the outcome of their pregnancy.

## 2. Materials and Methods

### 2.1. Experimental Overview—Animal Work

In total, 68 2–4-year-old Karagouniko breed ewes were included in the study, a part of which was performed during the summer months in central Greece. Ewes were allocated into two equal groups: Melatonin group (M) and Controls (C). Allocation was by using a random number generator to achieve complete randomisation.

After a 15-day acclimatization period, on the 1st June (D0), reproductive control was initiated. Specifically, a standard protocol was employed with intravaginal insertion of progestogen sponges (flugestone acetate 20 mg [Chronogest]; Intervet, Boxmeer, The Netherlands) from D0 to D14 and intramuscular eCG administration (dose rate: 300 UI; Gonaser, Laboratorios Hipra, Girona, Spain) on D14. Further, melatonin implants were also administered to ewes of M group on D0, D40, D80, and D120 (dose rate: 1 implant per ewe; Regulin, Ceva, Lisbourne, France) [24] subcutaneously at the base of the ear, by using an administration device. The first implant to each animal was administered on the left ear, with subsequent administrations performed on the right ear and again on the left and the right ears.

On D16, rams of known fertility were introduced into the ewes, at a ratio of 1:7 until D23 and were reintroduced on D38 until D48. Colored crayons fitted to rams and ewes found marked were considered to have been mated. On D50 and D84, pregnancy diagnosis was performed in all ewes by means of ultrasonographic examination (U/S). Ewes not found pregnant on D84 were excluded from the study. Finally, the number of lambs produced and their live bodyweight were recorded at birth.

Ewes were housed throughout the study. Starting on D0, the temperature and humidity outside and within the animal facilities were measured daily by means of a portable temperature data logger (HD 32.2, Delta OHM, Caselle di Selvazzano, Italy). For measuring conditions within the animal facility, the logger was placed on a table for 4 h daily between 12.00 and 18.00.

Similar health management practices (nutrition, vaccinations, anthelmintic treatments etc.) were applied in both groups. Rectal temperature and breathing rate were measured in nine animals in each group twice daily, at 12.00 and 18.00. Before lambing, blood samples were collected from nine ewes in each group (same animals as above) on D0, D23, D40, D80 and D120. Blood samples were collected from nine ewes in each group (same animals as above) immediately after lambing (L0) and then on L1, L2, L5, L10, and L40.

Milk yield measurements were performed on L5, L10 and L40 by using the method described in detail by Fthenakis and Jones [25,26].

Conditions prescribed by legislation of the European Union in relation to animal experimentation procedures (Council Directive 86/809/EEC) were met during this work (licence number of experimental works: 41-bio/exp-04, approvals by Veterinary Faculty: 37/2016 and 6/2018).

**Figure 1:**
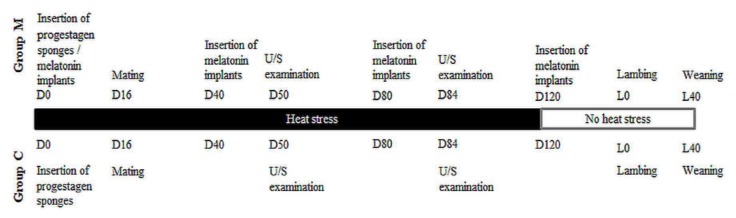
Illustrative timeline of the study.

A concise timeline of the study is presented in Figure 1.

### 2.2. Laboratory Examinations

Blood and colostrum/milk samples were appropriately prepared after collection and then stored at −20 °C. All measurements were performed within 3 months of collection.

Three redox biomarkers, namely total antioxidant capacity (TAC) as a crude index of the antioxidant potential of the samples, thiobarbituric acid reactive substances (TBARS) as a biomarker of lipid peroxidation, and reduced glutathione (GSH) as the most important intrinsic antioxidant molecule, were evaluated in all blood samples [27]. TAC was measured on the basis of the protocol of Janaszewska and Bartosz [28] as previously described [29]. In brief, 20 μL of each plasma sample was mixed with 10 mM sodium phosphate buffer pH = 7.4 (480 μL) and 0.1 mM 2,2-diphenyl-1-picrylhydrazyl radical (DPPH•) solution (500 μL). The mixture was incubated in the dark for 60 min at room temperature (RT), centrifuged (20,000× *g*, 3 min, 4 °C), and the absorbance was monitored at 520 nm. TAC was calculated on the basis of the mmol DPPH• reduced by the antioxidants present in the samples. TBARS were measured according to Keles et al [30] as previously described [31]. For the assay, 100 μL of plasma was mixed with 35% trichloroacetic acid (TCA) (500 μL) and 200 mM Tris-HCl pH = 7.4 (500 μL), the mixture was incubated for 10 min at RT and 1 mL of 2 M Na_2_SO_4_ and 55 mM of thiobarbituric acid (TBA) was added. Following a 45-min incubation at 95 °C, 1 mL of 70% TCA was added, the samples were centrifuged (15,000× *g*, 3 min, 20 °C) and the absorbance was monitored at 530 nm. The concentration of TBARS was calculated on the basis of the millimolar extinction coefficient of malonyldialdehyde (MDA) (156 L/mmol/cm). Finally, GSH was measured according to Reddy et al. [32] as previously described [33]. Briefly, 20 μL of erythrocyte lysate treated with TCA was mixed with 67 mM phosphate buffer (pH = 7.95) (660 μL) and 1 mM 5,5-dithiobis (2-nitrobenzoic acid) (DTNB) (30 μL), the mixture was incubated for 45 min in the dark at RT and the absorbance was measured at 412 nm. GSH concentration was calculated on the basis of the millimolar extinction coefficient of DTNB (13.6 L/mmol/cm). Hemoglobin concentration of erythrocyte lysate was measured using a commercially available kit. All measurements were performed in triplicate. In all cases, standard reagents were used (Sigma-Aldrich, Saint Louis, MO, USA).

### 2.3. Data Management and Analysis

#### 2.3.1. Temperature—Humidity Index

For the calculation of temperature humidity index (THI), which was proposed by Marai et al. and Habeeb et al. [34,35] as a useful index of heat stress severity, the below equation was applied:

THI = db °C – [(0.31 − 0.31 × RH) × (db °C − 14.4)],(1)

where db °C was the ‘dry bulb’ temperature provided by the logger (°C) and RH was the relative humidity provided by the logger.

For the interpretation of results, the average of the two values (12.00 and 18.00) was calculated (average daily THI) and then the standard provided by Marai et al. [34] was taken into account, as follows; < 22.20: no heat stress, 22.20–23.29: moderate heat stress, 23.30–25.59: severe heat stress, ≥ 25.60: extreme severe heat stress.

#### 2.3.2. Measures for Reproductive Performance

The following measures of reproductive performance were calculated [36].

- Mating rate: number of ewes marked by the ram during the two introductions into ewes/number of ewes exposed to the ram × 100.
- Pregnancy rate: number of ewes found pregnant at the ultrasonographic examination on D84/number of ewes exposed to the ram × 100.
- Lambing rate: number of ewes that lambed among those found pregnant at ultrasonographic examination on D84/number of ewes mated subsequently to application of reproductive control × 100.
- Total lambs per ewe: number of liveborn and stillborn lambs/number of ewes that lambed.
- Lamb bodyweight per ewe (kg): total weight (kg) of liveborn lambs/number of ewes that lambed.

#### 2.3.3. Statistical Analysis

Data were entered into Microsoft Excel and analysed using VassarStats (vassarstats.net., USA, 1998–2020). Basic descriptive analysis was performed. Statistical significance was set at *p* < 0.05.

THI, breathing rate and rectal temperature were compared within each group (M and C) between the daily time points (12.00 and 18.00) using the paired samples t-test. Moreover, breathing rate and rectal temperature were compared between groups M and C using the independent samples t-test. 

Association of THI with rectal temperature or breathing rate throughout the study in each animal was estimated by calculating Pearson’s correlation coefficient *r*. For this purpose, were taken into account every measurement throughout the study (9 animals of each group × 120 days = 1080 measurements). 

Pregnancy rate on D84 and lambing rate between the two groups were compared using the chi-square test. Number of total lambs born per ewe, mean bodyweight of lambs born and mean lamb bodyweight per ewe between M and C group were compared using the independent samples t-test.

The levels of the tested redox biomarkers (TAC, TBARS, GSH) in time-series blood samples [D0, D23, D40, D80, D120, lambing (L0), L1, L2, L5, L10 and L40] were compared within each group (M or C) and between the two groups (M and C) by using Analysis of Variance with Repeated Measures.

Correlations were performed at selected time points (D0, D23, D40, D80, and D120) in each animal between each redox biomarker (i.e., TAC, GSH, TBARS) and mean rectal temperature or mean breathing rate, respectively. For the correlations were taken into account every measurement throughout the study (9 animals of each group × 5 time points = 45 measurements). Firstly, the Pearson’s correlation coefficients *r* were calculated in each group separately. Then, the differences of *r* factors between groups M and C were evaluated separately for each redox biomarker and body temperature or breathing rate, respectively, by using Fisher *r* to *z* transformation. 

Moreover, correlations were performed in each animal between each redox biomarker assayed at D23 and the number of foetuses measured on D50 of the study. The redox status at D23 (i.e., on the 7th day of pregnancy) was evaluated because that day the level of administered melatonin has already been increased, as well as, the D23 coincides with the blastocyst formation. Firstly, the Pearson’s correlation coefficients *r* was calculated in each group separately. Then, the differences of *r* factors between groups M and C were evaluated separately for each redox biomarker and number of foetuses by using Fisher *r* to *z* transformation. 

Accordingly, milk production (L5, L10, and L40) was compared within each group (M or C) and between the two groups (M and C) by using the general linear model for repeated measures.

## 3. Results

### 3.1. Clinical Findings—THI during the Heat Stress Period

The results of the heat stress period are presented in 6 equally divided stages (S1-6), as mean ± standard error of the mean (SEM), in Table 1. 

Mean THI throughout the heat stress period was lower (*p* < 0.001) in midday (27.9 ± 0.2) compared with late afternoon (28.6 ± 0.2). Of the 240 occasions that measurements were performed, in 237 severe heat stress was recorded (Table 1). 

Clinical examination revealed that, throughout the studied period, the rectal temperature and the breathing rate were higher in the late afternoon examination. Mean rectal temperature for M group ewes was 39.21 ± 0.01 and 40.06 ± 0.01 °C and for controls 39.31 ± 0.01 and 40.12 ± 0.01 °C for the midday and the late afternoon measurement, respectively (*p* < 0.001 for all comparisons). Mean breathing rate for M group ewes was 60 ± 0 and 81 ± 1 breaths min^−1^ and for controls 61 ± 0 and 101 ± 1 breaths min^−1^ in the midday and the late afternoon examination, respectively (*p* < 0.001 for all comparisons). These differences were consistent during all 6 stages (S1–6, Table 1). Differences in average breathing rates and average temperatures throughout the study were also significant between M and C groups (*p* < 0.02 for all comparisons). Details are presented in Table 1. 

**Figure 2:**
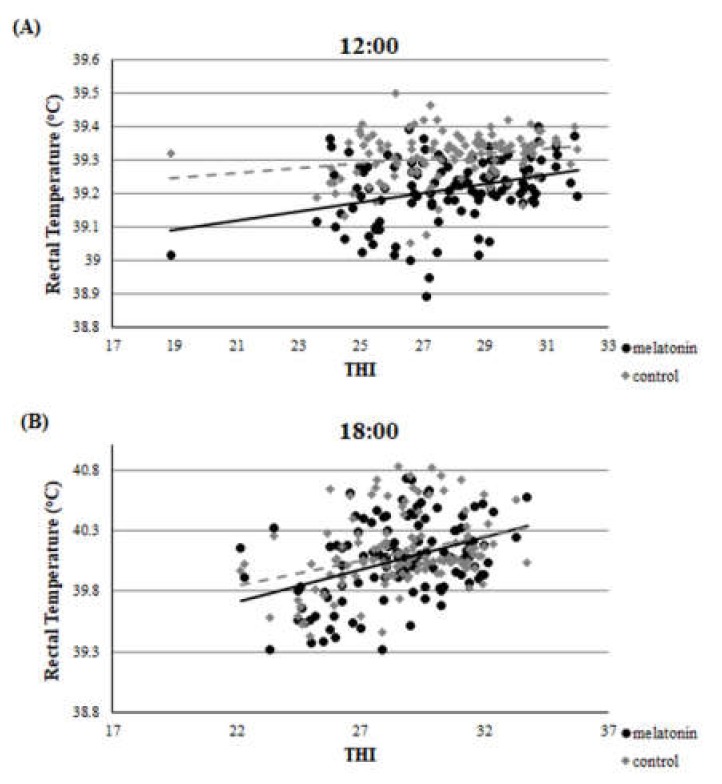
Correlation between THI and rectal temperature at the two measurements (**A**), (**B**), *r* > 0.22, *p* < 0.01.

**Figure 3:**
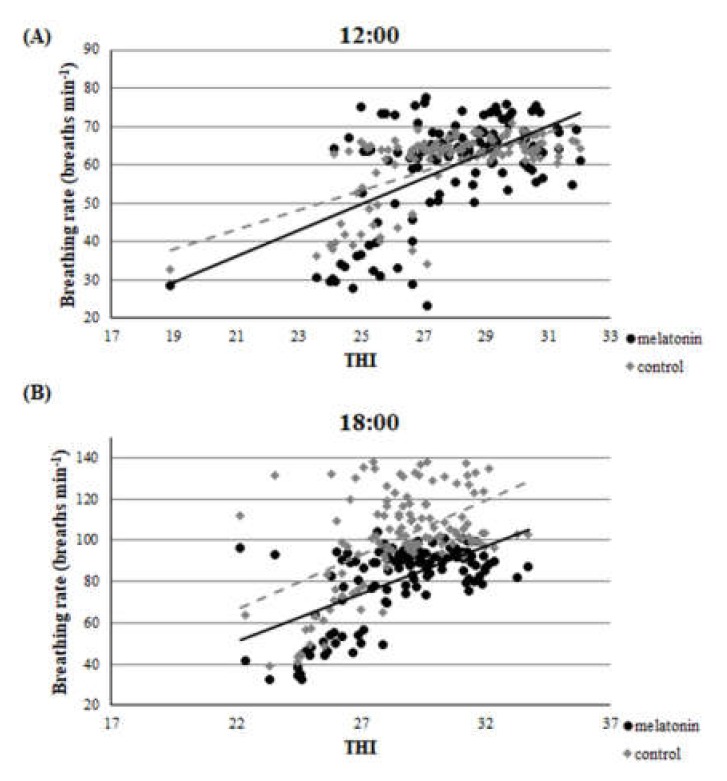
Correlation between THI and breathing rate at the two measurements (**A**), (**B**), *r* > 0.22, *p* < 0.01.

**Figure 4:**
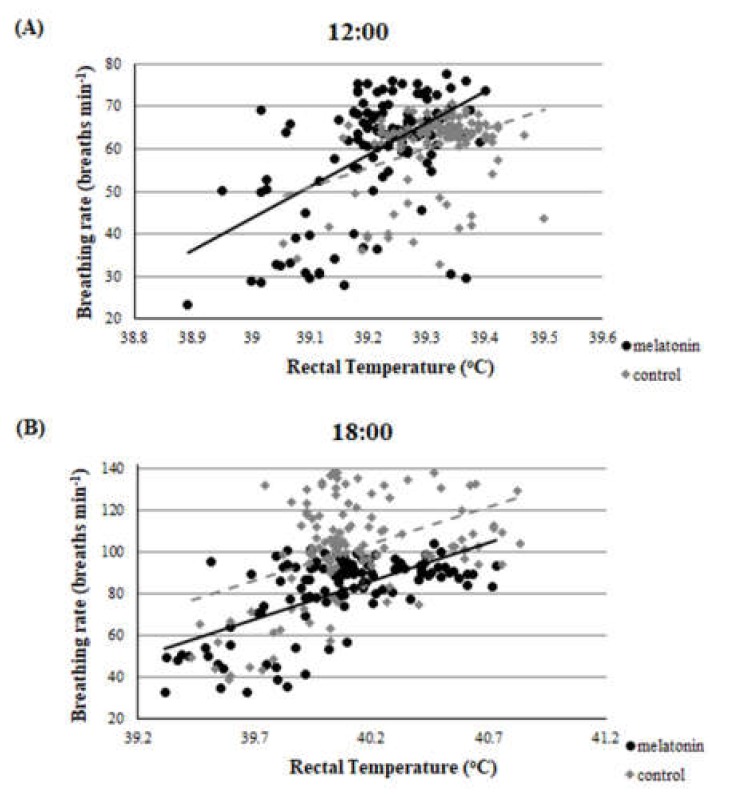
Correlation between rectal temperature and breathing rate at the two measurements (**A**), (**B**), *r* = 0.17 for 12:00 and *r* = 0.42 for 18:00.

Throughout the studied period, there was a clear correlation between THI and rectal temperature or breathing rate. This correlation was evident in both groups and in both measurements (*r* > 0.22, *p* < 0.01 for all measurements). Accordingly, there was a clear correlation between rectal temperature and breathing rate for ewes in M and C groups. (Figure 2, Figure 3 and Figure 4).

### 3.2. Reproductive Performance

Consequent to the application of reproductive control, 32 group M and 33 group C ewes were mated (*p* = 0.50). Pregnancy rate on D84 and lambing rate differed significantly between the two groups: 26/34 (76.5%) versus 19/34 (55.9%), for M and C group respectively (*p* = 0.035). All ewes lambed normally.

The number of total lambs born per ewe was higher in M than in C group ewes (mean ± SEM: 1.65 ± 0.10 versus 1.31 ± 0.09; *p* = 0.029). The mean bodyweight of lambs (irrespectively of single or multiple pregnancies) did not differ between groups (mean ± SEM: 4.03 ± 0.12kg versus 3.96 ± 0.12kg; *p* = 0.34); however, mean lamb bodyweight per ewe was higher in M than in C group (mean ± SEM: 6.67 ± 0.37 kg versus 5.21 ± 0.30kg; *p* = 0.003).

### 3.3. Redox Biomarkers 

**Figure 5:**
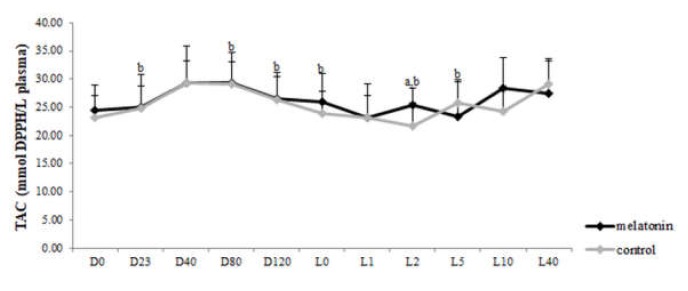
Total antioxidant capacity of ewes throughout the study. a: among the two groups, b: between consecutive time points in each group, *p* < 0.05 for differences.

**Figure 6:**
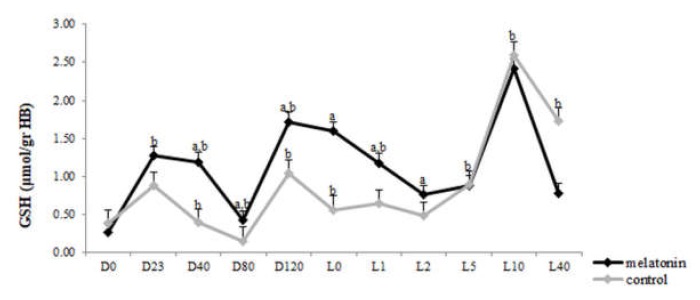
Glutathione levels of ewes throughout the study. a: among the two groups, b: between consecutive time points in each group, *p* < 0.05 for differences.

**Figure 7:**
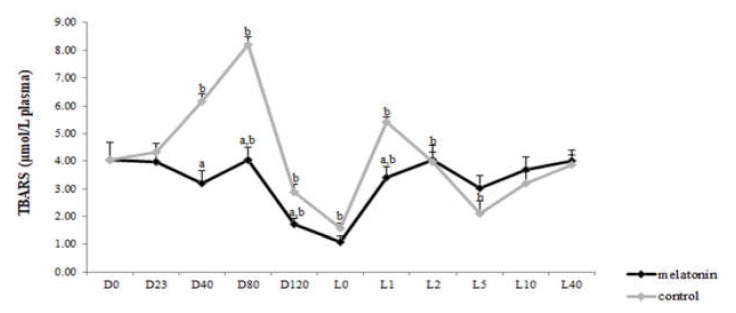
Lipid peroxidation in plasma of ewes throughout the study. a: among the two groups, b: between consecutive time points in each group, *p* < 0.05 for differences.

TAC concentration varied within time in each group (*p* < 0.043) (Figure 5). However, GSH levels showed variation not only within time in both groups (*p* < 0.05), but also between the two groups. Specifically, the melatonin treated ewes showed higher GSH levels (*p* < 0.004) than the control ones, starting from the D23 until L2 (Figure 6). TBARS varied within time only in control group (*p* < 0.0005). Also, TBARS were lower in the melatonin group compared with the control one, starting from D40 until L1 (*p* < 0.03) (Figure 7).

**Figure 8:**
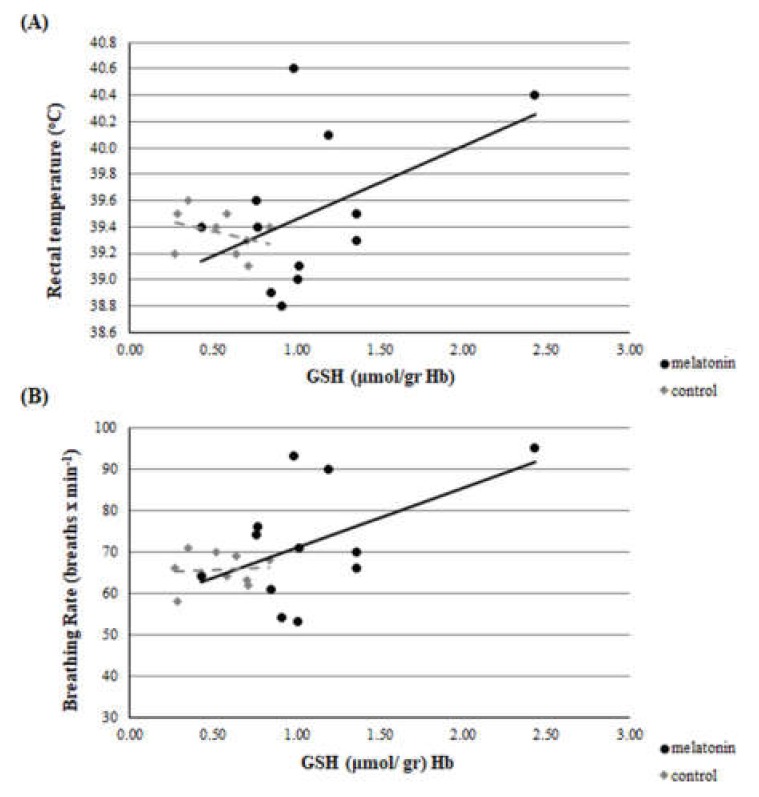
Correlation between GSH and rectal temperature (**A**) or breathing rate (**B**) on D23 [*z* = 1.66 and *p* = 0.048 for (**A**) and (**B**); *r* = 0.504, *p* = 0.047 for (**B**)].

**Figure 9:**
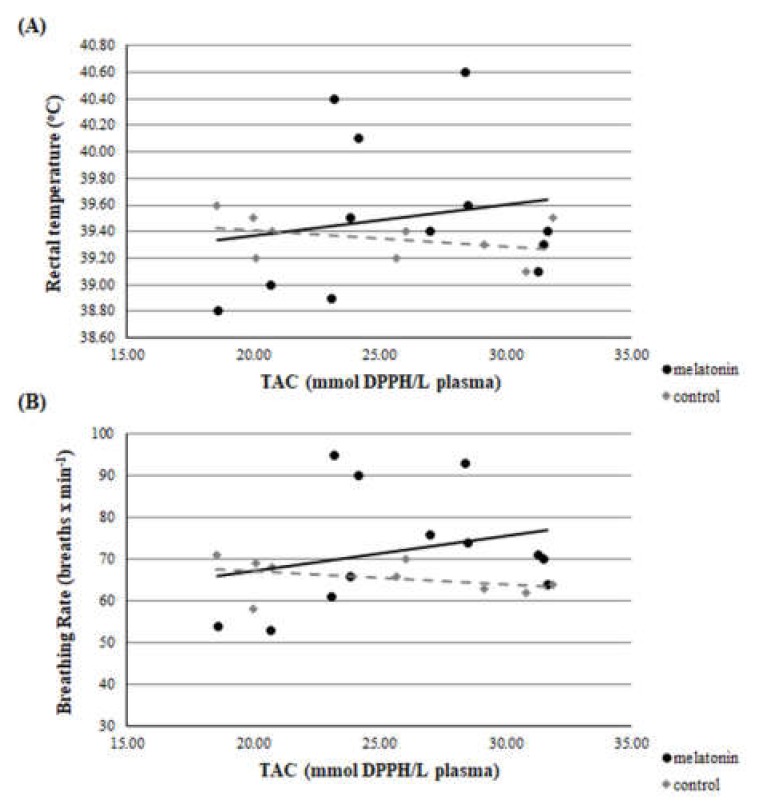
Correlation between TAC and rectal temperature (**A**) or breathing rate (**B**) on D23 (*p* > 0.135 for all comparisons).

**Figure 10:**
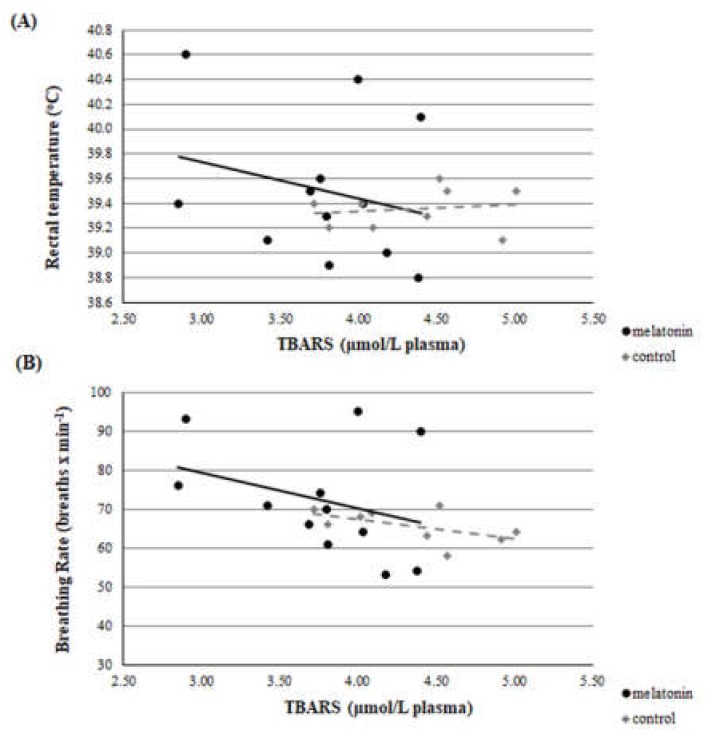
Correlation between TBARS and rectal temperature (**A**) or breathing rate (**B**) on D23 (*p* > 0.135 for all comparisons).

A clear correlation was found on D23 between GSH and breathing rate (*r* = 0.504, *p* = 0.047). Significant difference was found on D23 between GSH and rectal temperature, between the *r* of group M and group C (*z* = 1.66 and *p* = 0.048) (Figure 8). However, no correlation was found at D0, D40, D80, and D120 between each redox biomarker and body temperature or breathing rate, respectively, either in each group or between groups (*p* > 0.135 for all comparisons). Selectively, the correlations of D23 are presented in Figure 9 and Figure 10. 

**Figure 11:**
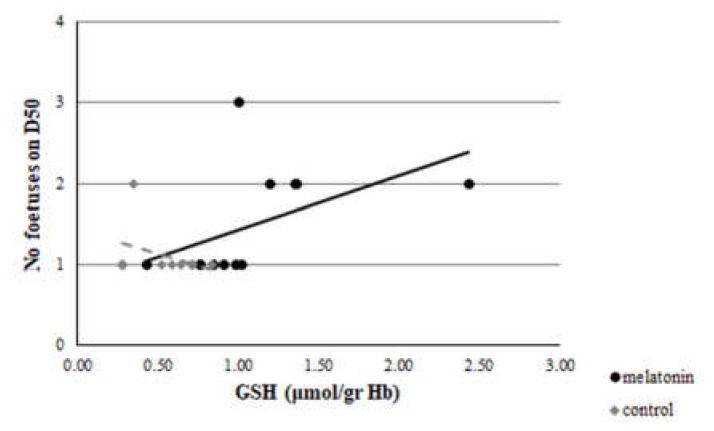
Correlation between GSH at D23 and number of foetuses measured on D50, *p* = 0.036.

**Figure 12:**
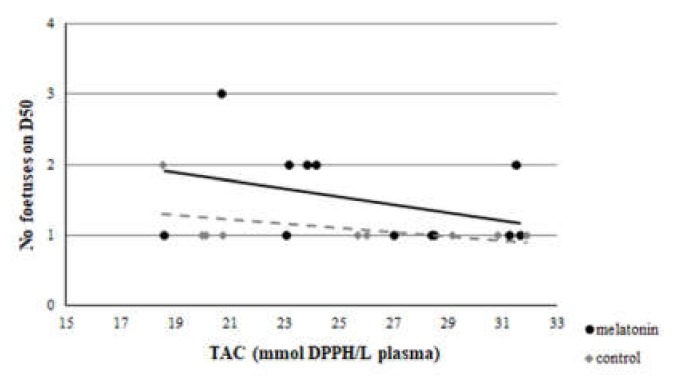
Correlation between TAC at day 23 and number of foetuses measured on D50, *p* > 0.146.

**Figure 13:**
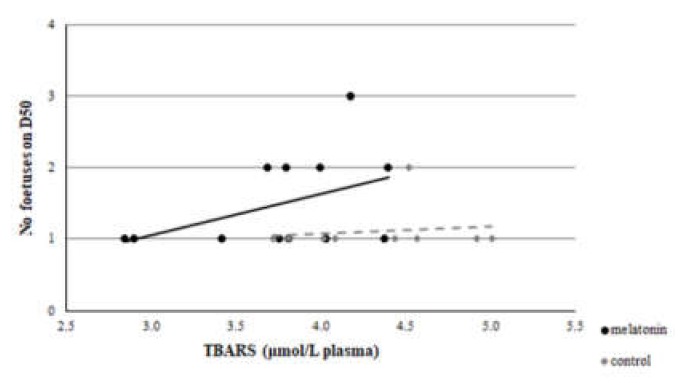
Correlation between TBARS at day 23 and number of foetuses measured on D50, *p* > 0.146.

A clear correlation was evident between GSH assayed at D23 and the number of foetuses measured on D50 of the study in the M group (*r* = 0.512, *p* = 0.044), but not in group C (*r* = -0.364, *p* = 0.17) (Figure 11). Also, a significant difference was found between the *r* of group M and group C (*z* =1.8 and *p* = 0.036). However, no correlation was found between TAC or TBARS assayed at D23 and the number of foetuses measured on D50 of the study in both groups (*p* > 0.146 for all comparisons) (Figure 12 and Figure 13). 

### 3.4. Milk Yield

**Figure 14:**
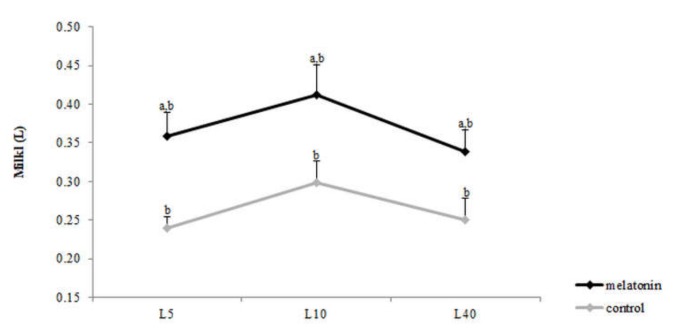
Milk yield 5, 10 and 40 days after lambing. a: among the two groups, b: within time, *p* < 0.031.

Milk production varied within time during puerperium in both groups (*p* < 0.031) and was higher in group M (*p* < 0.015) compared with C (Figure 14).

## 4. Discussion

This is the first study that evaluates the antioxidant effects of melatonin administered in pregnant ewes under heat stress conditions. It is indicated that the repeated administration of melatonin throughout the pregnancy period improves the redox status of heat stressed ewes. Melatonin treatment not only increased the mean number and bodyweight of lambs born per ewe but also led to higher milk production during the puerperium. The higher number of lambs born from the melatonin treated ewes is probably mediated through GSH, as it is supported by the clear correlation found between the number of foetuses measured on D50 and the levels of GSH assayed at the stage of blastocyst.

For the estimation of heat stress, many indicators have been proposed [12]. The primary indicator is THI [34], which was utilized in the present study. Apart from THI, as good indicators of heat stress in sheep can be assessed two physiological indices i.e., rectal temperature and breathing rate. Based on the latter three indicators, all participated ewes were imposed under severe heat stress until the 120th day of the study (i.e.,100th day of pregnancy), which is further amplified by the clear correlation found between THI and rectal temperature or breathing rate (Figure 2, Figure 3 and Figure 4). These physiological responses could be considered as thermoregulatory adjustments to maintain homeostasis [12]. When an animal is subjected to heat stress, the body expresses a number of different hematological, physiological, and behavioral traits and indicators to negate stress, including respiratory rate, heart rate, ruminal movement frequency, rectal and skin temperatures, and sweating rate [36]. 

In order to reduce the negative effects of heat stress on the redox status of pregnant ewes, melatonin was chosen due to its pleiotropic activity. Melatonin is an indoleamine with amphiphilic nature, which gives to the molecule the capacity to enter any cell compartment or body fluid. Due to its extensive solubility in lipids, melatonin easily passes by diffusion from the peripheral circulation to other fluids or cells and subcellular compartments. This lack of corn-partmentalization makes melatonin unique among antioxidants that are characteristically confined to one cellular compartment [37,38]. Comparatively, melatonin provides the greatest molecular protection from oxidative damage than other protective molecules [39]. 

Endogenous melatonin plays an important role in pregnancy, as well as, in parturition [40,41]. During pregnancy high levels of oxidative stress induce an increase inoxidative damage to lipids, which in some cases is inhibited by the antioxidative actions of pineal indoles [42]. Studies have proved that maternal plasma melatonin levels are elevated during pregnancy, reaching a maximum at term, and returning to basal levels immediately after labour [40]. The placenta expresses melatonin receptors and melatonin easily crosses it without being altered [40]. During normal pregnancy, melatonin acts as an antioxidant and appears to be essential for a successful pregnancy and for the regulation of development of foetal organs that are critical for the successful adaptation of the neonate to extrauterine life [43]. Matsuzuka et al. [14] showed that the alleviation of embryo death might be achieved by the administration of melatonin in mice.

In the present study, it was found that melatonin administration led to an improved pregnancy rate and a higher number of lambs born per ewe compared with the controls. These effects could be attributed either to selection/dominance of higher number of preovulatory follicles and/or to better luteal function and/or to better survival of fertilized oocytes (early embryos). Hansen [44] stated that heat stress disrupts the development and function of the oocyte. Heat shock seems to promote the generation of ROS, which damages the oocyte during the preovulatory period [14]. Both the effects of heat stress in vivo [4] and heat shock in vitro [5] were inverted by administration of antioxidants. Forcada et al. [45] found that melatonin implants enhanced ovulation rate in ewes on a low compared with high plane of nutrition. Additionally, it has been reported that administration of exogenous melatonin to sheep and goats improves levels of luteinizing hormone, luteal function, and embryonic survival [20,46,47]. The administration of melatonin seems to protect corpora lutea from ROS and has key roles in maintaining corpora lutea function in women [48] and in pinealectomized ewes [49].

Although the embryo is perishable to maternal heat stress, this sensitivity is decreased as the embryo is developing, and its coping mechanisms are developing, too [50]. Ealy et al. [51] found that exposure of lactating cows to heat stress at day 1 after oestrus, reduced the proportion of embryos that developed to the blastocyst stage at day 8 after oestrus. However, heat stress on days 3 (8–16 cells), 5 (morula), and 7 (blastocysts) had no effect on the proportion of embryos that developed to blastocysts at day 8. A similar pattern of developmental adjustment of thermal resistance has been described in sheep in in vivo conditions, as well [52]. Furthermore, the addition of melatonin to the ovary storage medium had beneficial effects on sheep oocyte development and embryo quality by reducing the oxidative stress caused by ROS and preventing the deterioration of oocytes [53,54,55]. Two high-affinity G-protein-coupled receptors, melatonin MT1 and MT2 receptors, are expressed in sheep oocytes, cumulus cells, and granulosa cells [56], and the presence of MT1 receptors was found in sheep blastocysts, confirming the direct impact of melatonin on embryo development [53]. Moreover, the exogenous melatonin treatment of bovine granullosa cells has been shown to suppress apoptosis through interaction of MT1 and MT2 in a time- and dose-dependent manner [57], leading to the conclusion that MT1 is a crucial factor for the survival of granulosa cells [58].This impact is further confirmed by the clear correlation that was found in the present study between the GSH assayed at D23 (i.e., on the 7th day of pregnancy when the blastocyst has already developed) and the number of foetuses measured on D50 (Figure 11).

Usually, the birth weight of twins or triplets is lower compared with single ones. Although higher number of lambs born per ewe in melatonin group, the body weight of these lambs at birth did not differ with controls. This is also supported by the fact that lamb bodyweight per ewe was higher in M group compared with controls. Probably, melatonin treatment of ewes under heat stress conditions led to better growth of embryos in multi pregnancies. 

The redox profile of ewes received melatonin implants was improved compared with controls. In particular, GSH levels were higher whereas TBARS concentration was decreased in the melatonin treated ewes after the second implant administration until 48 h post-partum. Sheep under heat stress show imbalance in their antioxidant system as reactive oxygen metabolites increase and antioxidant defenses hamper [11]. It has been, also, reported a heat stress-induced reduction in the level of TAC in humans which was reversed by exogenous melatonin [59]. In the present study TAC levels were high at parturition and soon after it, probably in order the animal to cope with the stress from parturition. 

The peculiar properties of melatonin are also supported in this study by the increase of GSH levels in the M group followed with simultaneous decrease of TBARS almost from the beginning of pregnancy until 48 h post-partum. Melatonin is a scavenger of both oxygen and nitrogen-based reactive species and promotes the production of endogenous antioxidants, such as GSH, which leads to the development of adaptations of both the maternal organism and embryo to heat stress conditions. Studies in mice have shown that maternal heat stress resulted in increased ROS levels in oviducts and embryos and reduced GSH content in recovered embryos [13,14,60]. GSH is required for induced thermotolerance in mice [61] and changes in redox status may be an important determinant for the development of induced thermotolerance.

The antioxidant effects of low concentrations of melatonin seems to be the outcome of the upregulation of glutathione peroxidase (GPx) gene expression and, thus, antioxidant activity, while at high concentrations its effects are attributed to direct radical scavenging action. It has been proposed that the ability of melatonin to up-regulate GPx involves both membrane and nuclear receptors. It appears that the stimulation of GPx by melatonin is the most consistent effect, while the effect on other enzymes is tissue-specific or conditional [37]. The antioxidant protection by melatonin includes, also, direct electron exchange reactions of the mitochondrial respiratory chain and the indirect effect of up-regulating several antioxidant enzymes and down-regulating pro-oxidant enzymes, as well as nitric oxide synthases [62,63,64]. Direct and indirect antioxidant functions of melatonin may explain the observed decrease of lipid peroxide end product (i.e., MDA) and the increase of GSH levels. Also, Reiter et al. [65], reported that pharmacological doses of melatonin (ranging from 0.1 to 4.0 mM) in a dose-response manner reduced lipid peroxidation products [MDA and 4-hydroxyalkenals (CHDA)] in rat brains in in vitro conditions. The aforementioned results are in accordance with the findings regarding the blood redox status assessed in the current study. 

The increase of milk production may be attributed to the antioxidant effects of melatonin during lactogenesis and/or to the higher number of lambs born. In the present study, melatonin last administration was performed 40 days before parturition, inferring that the melatonin concentration had been decreased to basal levels at parturition. The administration of melatonin implants to dairy cows at drying-off did not affect milk production albeit the moderate suppression of prepartum prolactin concentration [24]. Litter size is another important factor affecting colostrum composition and milk production. Twin-bearing ewes generally yielded more colostrum than single-bearing ewes, as well as, the capacity of milk production from the mammary gland depends on the number of lambs born per ewe [66,67].

Continuous effort has been made to assess the role of antioxidants in reproductive health and fertility rate under the risk of global warming [68]. In the present study, it is concluded that melatonin administration throughout pregnancy leads to the improvement of the redox status in heat-stressed ewes, as well as the pregnancy rate, number of lambs born, and milk yield during puerperium. Thus, melatonin could be used as an antioxidant regimen for inducing thermotolerance in sheep under heat stress conditions in order to improve their reproductive traits in regions with a hot climate, such as Thessaly, Greece.

## Figures and Tables

**Table 1:** Details of rectal temperature and breathing rate (mean ± SEM) in ewes under severe heat stress (total duration 120 days), presented in 6 equally divided stages.

Group	Time of Day	Stage of the Heat Stress Period					
		S1	S2	S3	S4	S5	S6
Rectal Temperature (°C)							
M	12.00	39.12 ± 0.02 ^a,m^	39.25 ± 0.02 ^a,m^	39.21 ± 0.02 ^a,m^	39.28 ± 0.01 ^a,m^	39.27 ± 0.01 ^a,m^	39.17 ± 0.02 ^a,m^
	18.00	39.85 ± 0.03 ^a,n^	40.18 ± 0.02 ^a^	40.10 ± 0.02 ^a^	40.18 ± 0.02 ^a,n^	40.24 ± 0.02 ^a^	39.84 ± 0.03 ^a^
C	12.00	39.30 ± 0.02 ^b,m^	39.33 ± 0.02 ^b,m^	39.30 ± 0.02 ^b,m^	39.32 ± 0.01 ^b,m^	39.35 ± 0.01 ^b,m^	39.27 ± 0.01 ^b,m^
	18.00	40.05 ± 0.03^b,n^	40.16 ± 0.02 ^b^	40.13 ± 0.02 ^b^	40.24 ± 0.02 ^b,n^	40.25 ± 0.03 ^b^	39.89 ± 0.03 ^b^
Breathing Rate (Breaths min^−1^)							
M	12.00	51.3 ± 1.1 ^c,o^	62.0 ± 0.7 ^c,o^	63.7 ± 0.9 ^c^	67.5 ± 0.6 ^c,o^	66.4 ± 0.6 ^c,o^	47.0 ± 1.1 ^c,o^
	18.00	69.8 ± 1.2 ^c,p^	87.3 ± 0.7 ^c,p^	87.0 ± 1.0 ^c,p^	91.0 ± 0.6 ^c,p^	90.8 ± 0.6 ^c,p^	61.6 ± 1.6 ^c,p^
C	12.00	57.0 ± 1.0 ^d,o^	66.3 ± 0.3 ^d,o^	61.8 ± 0.8 ^d^	63.4 ± 0.5 ^d,o^	63.8 ± 0.4 ^d,o^	51.8 ± 0.9 ^d,o^
	18.00	88.9 ± 1.1 ^d,p^	104.0 ± 0.8 ^d,p^	112.9 ± 1.4 ^d,p^	115.7 ± 1.1 ^d,p^	115.7 ± 1.3 ^d,p^	69.2 ± 1.8 ^d,p^
Thermal Index							
	12.00	27.9 ± 0.36 ^e^	29.7 ± 0.36 ^e^	28.1 ± 0.6 ^e^	28.5 ± 0.5 ^e^	27.3 ± 0.4 ^e^	25.9 ± 0.4 ^e^
	18.00	28.3 ± 0.38 ^e^	30.4 ± 0.57 ^e^	29.1 ± 0.5 ^e^	29.2 ± 0.4 ^e^	28.3 ± 0.4 ^e^	26.1 ± 0.4 ^e^

a–e within the same column: *p* < 0.05 for differences; m–p within the same column: *p* < 0.05 for differences.
